# The Association between Immune Subgroups and Gene Modules for the Clinical, Cellular, and Molecular Characteristic of Hepatocellular Carcinoma

**DOI:** 10.1155/2022/7253876

**Published:** 2022-08-31

**Authors:** Wenbiao Chen, Peng Zhu, Huixuan Xu, Xianliang Hou, Changchun Guo

**Affiliations:** ^1^Central Molecular Laboratory, People's Hospital of Longhua, The Affiliated Hospital of Southern Medical University, Shenzhen 518110, China; ^2^Research Center for Human Tissue and Organs Degeneration, Institute of Biomedicine and Biotechnology, Shenzhen Institutes of Advanced Technology, Chinese Academy of Sciences, Shenzhen 518055, China; ^3^Central Laboratory, Shenzhen Pingshan District People's Hospital, Pingshan General Hospital, Southern Medical University, Shenzhen 518110, China; ^4^Department of Clinical Medical Research Center, The Second Clinical Medical College of Jinan University, The First Affiliated Hospital Southern University of Science and Technology, Shenzhen People's Hospital, Shenzhen 518110, China; ^5^Department of Anesthesiology, Shenzhen Pingshan People's Hospital, Pingshan General Hospital, Southern Medical University, Shenzhen 518110, China

## Abstract

The heterogeneity of hepatocellular carcinoma (HCC) is related to immune cell infiltration and genetic aberrations in the tumor microenvironment. This study aimed to identify the novel molecular typing of HCC according to the genetic and immune characteristics, to obtain accurate clinical management of this disease. We performed consensus clustering to divide 424 patients into different immune subgroups and assessed the reproducibility and efficiency in two independent cohorts with 921 patients. The associations between molecular typing and molecular, cellular, and clinical characteristics were investigated by a multidimensional bioinformatics approach. Furthermore, we conducted graph structure learning-based dimensionality reduction to depict the immune landscape to reveal the interrelation between the immune and gene systems in molecular typing. We revealed and validated that HCC patients could be segregated into 5 immune subgroups (IS1-5) and 7 gene modules with significantly different molecular, cellular, and clinical characteristics. IS5 had the worst prognosis and lowest enrichment of immune characteristics and was considered the immune cold type. IS4 had the longest overall survival, high immune activity, and antitumorigenesis, which were defined as the immune hot and antitumorigenesis types. In addition, immune landscape analysis further revealed significant intraclass heterogeneity within each IS, and each IS represented distinct clinical, cellular, and molecular characteristics. Our study provided 5 immune subgroups with distinct clinical, cellular, and molecular characteristics of HCC and may have clinical implications for precise therapeutic strategies and facilitate the investigation of immune mechanisms in HCC.

## 1. Introduction

Hepatocellular carcinoma (HCC) is one of the most common malignant tumors with high incidence and poor prognosis [1]. The data from 2018 statistics indicated that HCC was the sixth most common malignancy and the fourth most common cause of cancer-related death in the world [2]. Current treatment of HCC is mainly surgical resection in the early stage, and radiotherapy and chemotherapy in the middle and late stages. In particular, advances in targeted and immunotherapy in the past decade have brought substantial benefits to patients. However, most diagnosed patients are already in advanced stages, with limited conservative treatment options [3, 4]. Moreover, the effect of immunotherapy varies greatly among different patients and different cancer types. Even for the most widely used anti-PD-1/PD-L1 and anti-CTLA4 therapies, only a small number of patients showed good response [3], indicating further research on the immune response in the tumor immune microenvironment.

HCC is considered to be one of the most heterogeneous tumors due to its comprehensive effect on genetic, metabolic, immune, infectious, and other factors [5]. The heterogeneity of hepatocellular carcinoma is the nonhomogeneity of tumor cells, stromal cells, and immunity, which is a barrier to the study of the molecular mechanisms of the disease and the improvement of clinical treatment. Therefore, it is an essential method to study the molecular and immune characteristics of HCC at the cellular level and to conduct molecular typing to resolve tumor heterogeneity [6]. Recently, Zhang et al. described the immune microenvironment of HCC at the level of single cells, and discovered a new immune cell subpopulation related to the mechanism of immune escape from tumors [7]. Yutaka et al. classified HCC into three immune subtypes with additional prognostic impact on histological and molecular classification [8]. The above studies demonstrated that the clarification of tumor heterogeneity can help understand the complexity of the immune microenvironment and assist in clinical treatment.

Studies have shown that the heterogeneity of HCC was associated with its own cellular and molecular characteristics and were affected by immune cell infiltration in the immune microenvironment and gene expression [9]. In this study, we divided HCC into novel molecular types, namely, 5 immune subgroups and 7 gene modules based on gene expression profiles and immune characteristics. Association analyses between immune subgroups and cellular and molecular characteristics showed that each of the 5 immune subgroups was connected with distinct gene expression, tumor immune infiltrating cells, gene function, and cellular composition. In addition, the 5 immune subgroups showed significant differences in clinical characteristics. The immune landscape further demonstrated the accuracy of molecular typing based on gene expression profile and immune characteristics, as well as the intrinsic molecular association of HCC heterogeneity.

## 2. Materials and Methods

### 2.1. Acquisition and Processing of HCC Datasets

The HCC datasets with the clinical information of experimental and validation cohorts were obtained from the Cancer Genome Atlas (TCGA) and gene expression omnibus (GEO) databases, respectively. For the experimental cohort, we used the TCGA GDC API to download RNA-Seq data and clinical follow-up information data of human hepatocellular carc-inoma (LIHC), including 424 samples. Next, the RNA-Seq data of TCGA were preprocessed in the following steps: (1) removing samples without clinical data; (2) getting rid of data of normal tissue samples; (3) genes with transcript per million (TPM) expression equal to 0 in more than 50% of the samples was removed; (4) the expression profile of immune cell-related genes was retained and were subjected to log2 (TPM+1) transformation. A total of 371 samples in the TCGA cohort met the requirements and were selected in this study. For experiment cohorts, we downloaded 433 samples from GSE36376 and 488 samples from GSE14520. Then, the data were preprocessed in the following steps: (1) normal tissue sample data were removed; (2) taking out probes with null gene test value; (3) mapping these probes into human genes; (4) preserving the expression profile of immune cell-related genes. Ultimately, we obtained 240 and 225 samples from GSE36376 and GSE14520, respectively, for verification analyses.

### 2.2. Immune-Related Genes Selection

The following genes were collected as immune-related genes for subsequent analysis through literature retrieval and data mining. First, we collected immune cell-specific genes derived from the GEO database. Second, the costimulatory and coinhibitory receptors display great diversity in expression, structure, and function, and determine the functional outcome of T cell receptor (TCR) signaling which was obtained from Chen et al. literature [10]. Third, the genes of cytokines and their corresponding receptors were downloaded from the Kyoto Encyclopedia of Genes and Genomes (KEGG) database [11]. Fourth, the genes that participated in antigen processing and presentation were collected from the immunological assay data of ImmPort [12]. Fifth, we also took several immune-related genes that were related to at least one of the abovementioned immune genes into consideration, which to some extent, may have a potentially complex relationship with the tumor immune microenvironment. In total, 1989 immune-related genes met the inclusion criteria and were used for molecular typing analyses.

### 2.3. Immune Subgroups and Gene Modules Analyses

Based on the expression data of 1989 immune-related genes, a consistency matrix was constructed by consensus cluster-plus to obtain immune subgroups of samples. We used the partition around medoids (PAM) algorithm and Euclidean distance and went through 500 bootstraps, with each bootstrap including 80% of the training set of patients. The clustering number was set from 2 to 10, and the optimal classification was determined by calculating the consistency matrix and consistency cumulative distribution function. We also conducted the identical methods with the same settings and parameters to identify gene modules, in addition to using 1-Pearson correlation as a distance metric. Further, the function of immune-related genes in terms of Gene Ontology (GO) was analyzed by the Bioconductor *R* package. We used the average expression level of all the genes in the module to define the gene module score. The association between gene module score and molecular signatures was assessed by Spearman correlation analysis. The reproducibility of immune subgroups was verified in the GSE36376 dataset by quantitatively measuring the consistency of subgroups between the experimental and validation cohorts.

### 2.4. Analyses of the Association between Immune Subgroups and Cellular, Molecular, and Clinical Characteristics

The association between immune subgroups and cellular and molecular characteristics was assessed by analysis of variance. Four clustered immune cell types and 22 immune cell types that were identified by CIBERSORT arithmetic were used to analyze the composition of the immune cells [13]. These four clustered immune cells are referred to as total mast cells (the total percentage of activated mast cells and the percentage of resting mast cells), total macrophages (the total percentage of M0, M1, and M2), total lymphocytes, and total dendritic cells (the total percentage of activated dendritic cells and the percentage of resting dendritic cells), respectively. Stromal fraction is the number of tumor purity minus matrix fraction obtained by ABSOLUTE methods [14]. The polarization status of CD4+ T cells, including Th17, Th1, and Th2 was calculated by Bindea and colleagues' methods [15]. The leukocyte ratio was estimated from a mixed model containing 2,000 methylated probes, with the largest difference between pure leukocyte cells and normal tissues. By measuring the not a part of the polyclonal tumor genome, the intratumor heterogeneity was determined via ABSOLUTE methods [14]. Image-based tumor-infiltrating lymphocytes (TIL), were defined as the number of 50 × 50 micron areas being positive for tumor-infiltrating lymphocytes, which exceeded the total number of areas on the histological image. In addition, seven molecular characteristics, including cytolytic, macrophage regulation, IFN-*γ* response proliferation, lymphocyte infiltration, TGF-*β* response, and wound healing were defined in the corresponding literature, respectively. Aneuploidy scores were the sum of the amplified or deleted chromosome arms that were calculated by the ABSOLUTE algorithm [14]. The number of altered segments represented the proportion of bases that deviate from baseline ploidy. Homologous recombination defect score is a summary of 3 independent indicators of the genomic scar with allelic imbalance in subtelomeric regions. The diversity of the TCR and BCR was estimated using MiTCR arithmetic. Based on the HLA types obtained by OptiType methods from the RNA sequence [15], the SNV or Indel neoantigen was identified using NetMHCpan methods [16]. Moreover, several immune-related genomics signatures were incorporated into this study. “The tumor mutation burden” was defined as the rate of nonsilent mutation multiplied by 100. For the association between immune subgroups and clinical characteristics, the Kaplan–Meier method was used for survival analysis, and the log-rank test was used for comparison. Univariate Cox and multivariable Cox regression were conducted to evaluate the prognostic value of immune subgroups using the clinical signatures as concomitant variables.

### 2.5. Depiction of Immune Landscape

We used a graph-based learning approach for dimensionality reduction analysis to reveal the internal structure of the immune system and visualize the distribution of individual patients. This analysis addressed high-dimensional gene expression data into a tree structure in a low-dimensional space by preserving local geometric information, which has previously been used to model cancer progression and define the trajectory of development using large and single-cell gene expression data [17]. We extended our analysis to immune gene expression profiling, which may therefore, reflect relationships between patients in a nonlinear manifold and complement the discrete immune subtypes. Then, we used the different color counterparts of plot cell trajectory methods, with different colors corresponding to different immune subgroups, to visualize the immune landscape.

## 3. Results

### 3.1. Identification of Immune Subgroups and Gene Modules

**Figure 1:**
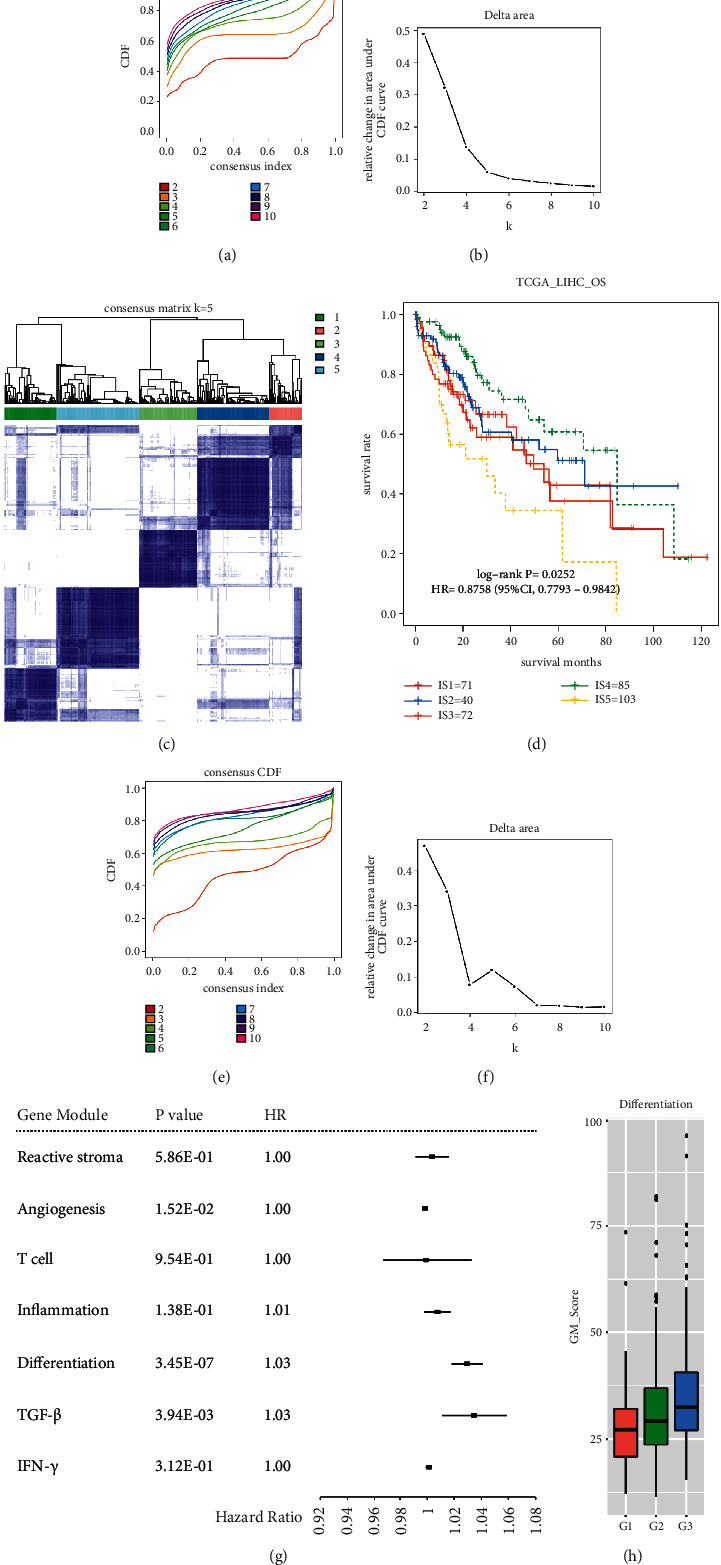
Identification the immune subgroups and gene modules in HCC. (a) The CDF curve of the samples for immune subgroups. (b) The CDF delta area curve of the samples for immune subgroups. Delta area curve of consensus clustering, indicating the relative change in area under the CDF curve for each category number k compared with k-1. (c) The clustering heatmap of samples when consensus *K* = 5. (d) KM curve of prognosis of 5 immune subgroups. (e) The CDF curve of the samples for gene modules. (f) The CDF delta area curve of the samples for gene modules. Delta area curve of consensus clustering, indicating the relative change in area under the CDF curve for each category number *k* compared with k-1. (g) Univariate Cox analysis of gene modules. (h) The correlation between the expression of gene module 5 and histological grade.

Consensus clustering was performed on 371 HCC samples from TCGA based on the gene expression profiles of 1900 immune-related genes to identify appropriate molecular typing. According to the accumulative distribution function (CDF), the optimal number of clusters was determined by observing the CDF delta area curve. It could be seen that cluster results were relatively stable when cluster selection was 5 (Figure 1(a)-1(b)). Finally, we selected *k* = 5 to obtain five immune subgroups (IS1 to IS5) (Figure 1(c)). Using clinical follow-up information on HCC samples, we found that there were significant prognostic differences among 5 immune subgroups (Figure 1(d)). IS4 was associated with the best prognosis, whereas IS5 had the worst prognosis. Consistent with the experimental cohorts, the prognostic outcome was well verified in the GSE14520 database (Figure S1). Due to HCC being mainly caused by hepatitis B virus (HBV) infection, we divide HCC into HBV-related and non–HBV-related HCC groups. Of note, the results were similar to the experimental cohort in the survival analysis both in two groups (Figure S2). These results determined the accuracy of five immune subgroups. Besides, the immune subtype was an independent prognostic factor in HCC according to univariate and multivariate Cox regression analysis (Table S1). Next, by similar methods, we identified 7 gene modules (GM) (Figure 1(e)–1(f)), and several gene modules were significantly correlated with HCC prognosis. In correspondence with the previous studies, the high scores in TGF-*β* and differentiation predicted poor outcomes (Figure 1(g)). The verification in validation cohorts also revealed that the differentiation module presented a poor prognosis of HCC (Table S2). The annotation of gene module 5 (differentiation) was associated with the histological grade that higher grade had a higher differentiation score (Figure 1(h)). These above outcomes suggest that HCC patients can be classified into different molecular subgroups based on immune characteristics and gene expression, with distinct clinical relevance.

### 3.2. Association Analysis between Immune Subgroups and Gene Modules

**Figure 2:**
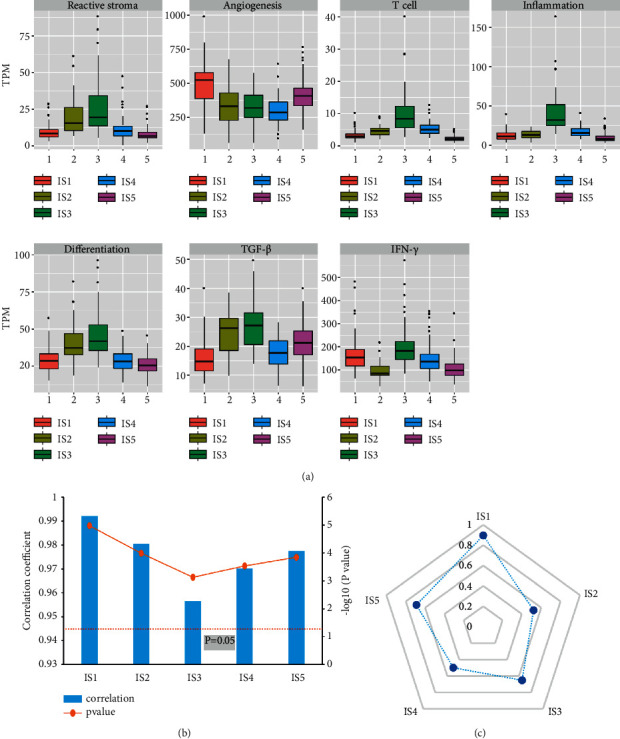
The association of immune subgroups and gene modules. (a) The distribution of 7 gene modules patterns among 5 immune subgroups. (b) Correlation of average scores across immune subgroups of the experimental and validation cohorts. (c) In-group proportion assess the similarity and reproducibility of the proposed immune subgroups between experimental and validation cohorts.

The heatmap of association between immune subgroups and gene modules revealed that each immune subgroup was connected with distinct expression patterns of seven gene modules (Figure S3). IS5 was related to the lowest expression in the gene modules of immune characteristics of T cells, inflammation, and IFN-*γ*, as well as the tumorigenesis characteristic of reactive stroma and differentiation, suggesting that IS5 was an immune cold type (Figure 2(a)). IS5 characteristic was followed by IS1, which also had the low expression in the gene modules of the immune characteristic of T cell, inflammation, reactive stroma, and differentiation, but with high expression of genes of IFN-*γ* and angiogenesis (Figure 2(a)). On the contrary, IS3 had the highest expression in the immune-related gene modules and the tumorigenesis-related gene modules, which was defined as an immune hot type (Figure 2(a)). Unlike IS5 and IS3, we found that IS4 had high expression of T cells, inflammation, and IFN-*γ*, and low expression of tumorigenesis modules like reactive stroma, angiogenesis, differentiation, and TGF-*β*, implying that IS4 belongs to both immune hot and antitumorigenesis types (Figure 2(a)). The above results were consistent with their prognosis that IS4 with the immune hot and antitumorigenesis types had the longest overall survival while IS5 with the immune cold type had the worst prognosis (Figure 1(d)). Next, we validated our findings using an independent GSE36376 dataset, and the results represented a high linear correlation of the expression level of immune subgroups between experiment (TCGA) and validation (GSE36376) cohorts with a mean value of Pearson correlation coefficient of 0.98 (Figure 2(b)). The intra-group correlation coefficients of IS1 to IS5 were 0.611, 0.52, 0.6, 0.559, and 0.843, respectively, suggesting a moderate to good agreement between the experiment (TCGA) and validation (GSE36376) cohorts in immune subtypes (Figure 2(c)).

### 3.3. Association Analysis between Immune Subgroups and Cellular and Molecular Characteristics

**Figure 3:**
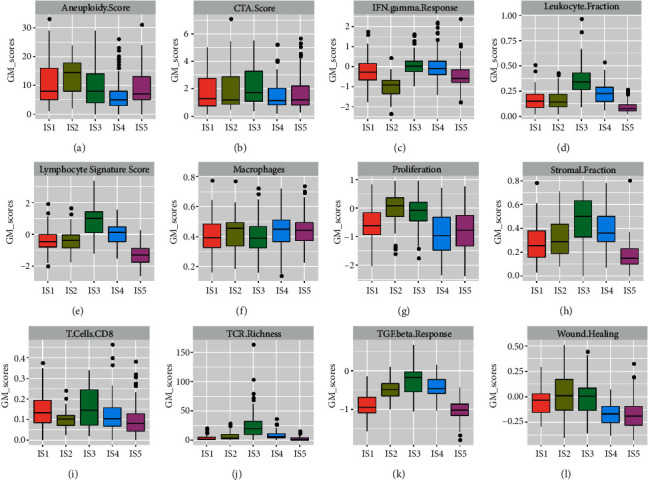
The association among immune subgroups and cellular, and molecular characteristics.

The heterogeneity of hepatocellular carcinoma (HCC) was related to immune cell infiltration and genetic aberrations in the tumor microenvironment and was affected by cellular and molecular characteristics, thus, we assessed the association between immune subgroups and several defined molecular and cellular characteristics (Figure 3). IS5 was associated with the lowest level of leukocytes (Figure 3(d)), lymphocytes (Figure 3(e)), T cell receptor (TCR) (Figure 3(j)), CD8 T cells (Figure 3(i)), follicular helper T cells (Figure S6G), memory B cells (Figure S5A), and resting dendritic cells (Figure S5E), which is in line with the immune cold type. However, IS5 was connected to a high degree of gene mutation, such as aneuploidy, fraction alteration, and proliferation. The IS1 that closely followed subtype IS5 also had the low expression of leukocytes (Figure 3(d)), and lymphocytes (Figure 3(e)), whereas IS1 had higher enrichment of TCR (Figure 3(j)), CD8 T cells (Figure 3(i)), follicular helper T cells (Figure S6G), and memory B cells (Figure S5A). IS3 was defined as immune hot type, IS3 had an increased level of leukocytes (Figure 3(d)), lymphocytes (Figure 3(e)), CD8 T cells (Figure 3(i)), TCR (Figure 3(j)), dendritic cells (Figure S5E), Th1 cells (Figure S5N), as well as high expression of IFN-gamma (Figure 3(c)), proliferation (Figure 3(g)), stromal fraction (Figure 3(h)), TGF-beta (Figure 3(k)), and wound healing (Figure 3(l)). Unlike the immune subgroups of IS5 and IS3, the favorable prognosis of IS4 represented the friendly and supportive immune profile. IS4 was enriched in immune cell infiltration of leukocytes (Figure 3(d)), lymphocytes (Figure 3(e)), CD8 T cells (Figure 3(i)), and CD4 T cells (Figure S5L). In contrast, IS4 was associated with a low degree of gene mutation of aneuploidy (Figure 3(a)), fraction altered (Figure S4C), proliferation (Figure S3G), stromal fraction (Figure 3(h)), TGF (Figure 3(k)), and wound healing (Figure 3(l)). The above results suggest that immune subgroups are associated with genomic and immune characteristics.

### 3.4. Depicting Landscape of Immune Subgroups

**Figure 4:**
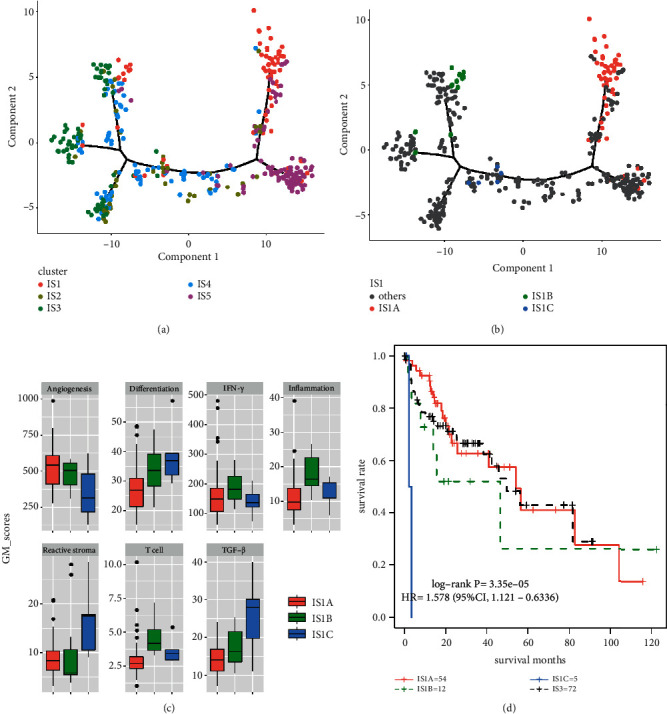
The depiction of immune landscape of HCC. (a) The trajectory of development of immune subgroups based on immune landscape. Each color represented the previously defined immune subtype, and each dot represented a patient. (b) The trajectory of development of 3 subtypes from IS1 based on immune landscape. Each color represented the previously defined immune subtype, and each dot represented a patient. (c) The distribution of 7 gene modules patterns among 3 subtypes from IS1. (d) KM curve analysis of prognosis of 3 subtypes from IS1.

To facilitate visualization and reveal the underlying structure of individual sample distribution, we performed a dimensionality reduction method [17] based on graph learning to depict the landscape of immune subgroups. This analysis placed a single sample into a graph with a sparse tree structure and defined the immune landscape of HCC. The position of a sample within them represented the overall characteristics of the immune microenvironment of the corresponding immune subgroups. For instance, IS3 and IS5 representing the immune hot and cold type, respectively, were distributed at opposite ends of the horizontal axis of the immune landscape (Figure 4(a)), whereas the friendly and supportive immune profile of IS4 was distributed on the middle of the horizontal axis of the immune landscape (Figure 4(a)). Due to the IS1 closely following subtype IS5, the immune landscape showed that the position of IS1 was close to IS5 (Figure 4(a)). In addition, immune landscape analysis further revealed significant intraclass heterogeneity within each immune subgroup. For example, we can further divide IS1 into 3 subtypes (IS1A-C) according to their position in the immune landscape (Figure 4(b)), and each subtype showed a specific pattern of immune expression (Figure 4(c)). Similar results were also identified in IS3 (Figure S7), IS4 (Figure S8), and IS5 (Figure S9). Particularly, the 3 subtypes of IS1 showed different gene expression patterns and survival times (Figure 4(c)-4(d)). IS1C had the worst prognosis and was abundantly enriched in tumorigenesis of angiogenesis, differentiation, react stroma, and TGF−*β*, as well as the low expression of immune signatures including IFN−*γ*, inflammation, and T cells (Figure 4(c)). On the contrary, the gene expression patterns and prognosis of IS1A were opposite to that of IS1C (Figure 4(c)). In the GSE14520 validation cohort, we also found similar prognostic trends in these subtypes (Figure S10). The above results further demonstrated the reliability of the immune subgroups.

## 4. Discussion

HCC is highly heterogeneous, and even the same HCC tissue is obviously different [17]. Current clinical and pathological classification cannot accurately assess the heterogeneity of HCC, and therefore, could restrict immunotherapy [17, 18]. Here, we performed multicohort retrospective analyses on independent experimental and validation cohorts to identify novel molecular typing of HCC according to genetic and immune characteristics. The study found that HCC patients could be segregated into 5 immune subgroups and 7 gene modules, and the association between immune subgroups and gene modules revealed that each immune subgroup had distinct patterns in cellular, molecular, and clinical characteristics. Our study depicted the immune and genetic characteristics of HCC based on molecular typing, which contributed to the understanding of the immune landscape of HCC and may have had substantial help in clinical implications for HCC.

The characteristic of being hot or cold of a tumor is determined by the information of the cancer cell itself, which is distinguished mainly by the number of immune cells in the tumor microenvironment [19]. In particular, a tumor was not a large group of tumor cells gathered irregularly but a complex microenvironment system, in which there were not only cancerous cells but also many symbiotic normal cells, immune cells, etc., such that immune cells and tumor cells interacted with each other to affect the outcome of tumor cells [20]. It was reported that using immune-checkpoint inhibitor therapy on patients with hot tumors helped activate existing immune cells to destroy cancer cells. However, for cold tumors, immunotherapy is ineffective because the immune cells do not recognize the tumor cells and it was useless to activate the immune system [21]. In this study, we found that HCC could be segregated into 5 immune subgroups with significantly different molecular, cellular, and clinical characteristics, particularly, IS5 and IS3 were revealed as immune cold and hot types, respectively. Consistent with the previous research, the immune cold type of IS5 had the worst prognosis, suggesting the inactivity of the immune system in killing tumor cells [22]. However, the immune hot type of IS1 had a better survival prognosis than IS5. Moreover, the hot or cold of a tumor was not the only factor that determined the effectiveness of immunotherapy, unlike previous research that only divided tumor cells into the immune cold and hot types [23]. Our analysis further stratified HCC into 5 immune subgroups, and IS4 with the best prognosis was characterized by immune activity and antitumor genesis. Thus, IS4 has both immune hot and tumor-fighting properties. In addition, recent studies have shown that the classification of cold and hot tumors is mainly based on the number of T cells in the tumor microenvironment [24]. And in the immune hot type, there were a lot of T cells around cancer, but the tricky tumor cells could disguise themselves to evade the inhibition of T cells [25], which may explain why the prognosis in the immune hot type of IS3 was not the best, even though IS3 had the highest expression of T cells. Our study may provide more detailed molecular characteristics for the classification of HCC; that is, finding the characteristics of immune activation and tumor suppression between cold and hot tumors may be more helpful for immunotherapy.

The heterogeneity of HCC is related to immune cell infiltration and genetic aberrations in the tumor microenvironment [26]. Therefore, we found that 5 immune subgroups represented distinct cellular and molecular characteristics. The HCC of IS5 was determined by the lowest levels of immune cell infiltration and a high degree of gene mutation. In comparison, IS3 had increased immune cell infiltration and high expression of IFN. Unlike extreme IS3 and IS5, IS4 was enriched in immune cell infiltration and was associated with a low degree of gene mutation. Accordingly, IS5, IS3, and IS4 had the worst, intermediate, and best prognosis, respectively (Figure 1(d)). Besides, the survival differences among 5 immune subgroups were independent of clinical factors. These results suggest that the immune subgroups based on immune profiling may be a key determinant of HCC molecular type prognosis and may be incorporated into future biomarker-based risk stratification strategies for individualized therapy. Moreover, the efficacy of immune typing has been fully validated in independent data sets, including independent data of HCC, HBV-related, and non-HBV-related HCC.

There have been a large number of studies on the molecular typing of HCC, and molecular typing based on genes, immunity, metabolism, and noncoding RNA has greatly supplemented the current clinicopathology [27–29]. Yutaka et al. found that the immune microenvironment of HCC can be divided into immune-high, immune-mid, and immune-low, which has additional prognostic effects on the histological and molecular typing of HCC [8]. Also, immune-high was characterized by increased immune cell infiltration with independent positive prognosis [8], which was consistent with our results that the subtype enriched in immune cell infiltration was associated with favorable survival. Traditionally, molecular-based typing was used to develop predictive and prognostic biomarkers, which require multidimensional analytical approaches to study gene expression profiles and to understand clinical treatment responses and clinical outcomes [30]. Here, to facilitate visualizing and revealing the underlying structure of individual patient distribution, we applied graph learning-based dimensionality reduction techniques to molecular typing. This approach has previously been used to model cancer progression and define developmental trajectory using large volumes of single-cell gene expression data, allowing for a more intuitive representation of the dynamic characteristics of molecular typing using the immune landscape platform [17]. In addition, when combined with immune cell dynamics, molecular characteristics, and clinical information, molecular typing features can be more comprehensively displayed [31]. We found that the immune hot type of IS3 and the immune cold type of IS5 were distributed at opposite ends of the horizontal axis of the immune landscape, whereas the supportive immune profile of IS4 on the middle of the horizontal axis of the immune landscape. These dynamic manifestations indicate that the HCC molecular typing in this study has significantly different characteristics. Although the immunological landscape generally summarizes immunological subtypes based on cluster analysis [32], it does not fully reveal intracluster heterogeneity and potential clinical relevance. Thus, we further used immune landscapes to reveal significant intracluster heterogeneity in each immune type. We found that certain subtypes seem to be more diverse and heterogeneous. For example, IS1C with the worst prognosis presented highly expressed gene modules of differentiation, TGF-*β,* and reactive stoma, which havesimilar characteristics to IS3, but they are located in different positions on the horizontal axis of the immune landscape map and have different prognostic characteristics. These results suggest our classification of 5 immune subgroups in HCC was not yet the most detailed, and the heterogeneity of HCC required further detailed classification to achieve the most accurate personalized diagnosis and treatment.

In conclusion, we identified and validated 5 immune subgroups with distinct molecular, cellular, and clinical characteristics. The immune landscape of 5 immune subgroups further demonstrated the accuracy of molecular typing based on immune characteristics, as well as the intrinsic molecular association of HCC heterogeneity. Our study may provide new insights into the immune characteristics of HCC and may promote the application of novel immune subgroups in the clinical management of HCC.

## Data Availability

The datasets analyzed in this study could be found in GSE36376 at https://www.ncbi.nlm.nih.gov/geo/query/acc.cgi?acc=GSE36376 and in GSE14520 at https://www.ncbi.nlm.nih.gov/geo/query/acc.cgi?acc=GSE14520.

## Supplementary Materials

Supplementary Materials Supplementary Materials. Figure S1. The prognosis of 5 immune subgroups was verified in validation cohort by KM analysis. Figure S2. The prognosis of 5 immune subgroups was verified in HBV-related and non-HBV-related HCC groups by KM analysis. Figure S3. The immune subgroups and gene modules in the HCC of the experimental cohort. Patients were arranged based on the predicted immune subgroups. Genes were ordered according to gene modules. Figure S4. The molecular characteristics of the immune subgroups. Figure S5. The distribution of 15 inferred immune compositions across 5 immune subgroups. Figure S6. The distribution of 7 inferred immune compositions across 5 immune subgroups. Figure S7. The depiction of the immune landscape of HCC from IS3-5. (A) The trajectory of development of 3 subtypes from IS3 based on the immune landscape, and (B) the distribution of 7 gene module patterns among 3 subtypes from IS3. Figure S8. The depiction of immune landscape of HCC from IS4. (A) The trajectory of development of 3 subtypes of IS4 based on the immune landscape, and (B) the distribution of 7 gene module patterns among 3 subtypes of IS4. Figure S9. Figure S9. The depiction of the immune landscape of HCC from IS5. (A) the trajectory of development of 3 subtypes from IS5 based on the immune landscape, and (B) the distribution of 7 gene module patterns among 3 subtypes from IS5. Figure S10. The verification by KM analysis of 3 subtypes from IS1 on validation cohort. Table S1. Univariate and multivariable Cox regression analyses of overall survival across clinical index and immune subtypes. Table S2. Validation of gene modules via univariate Cox analysis.
