# Supplementary figures and images for: The Association between Immune Subgroups and Gene Modules for the Clinical, Cellular, and Molecular Characteristic of Hepatocellular Carcinoma
Source: J Oncol. 2022 Aug 31;2022:7253876. doi: 10.1155/2022/7253876 (PMC9452932; doi:10.1155/2022/7253876)

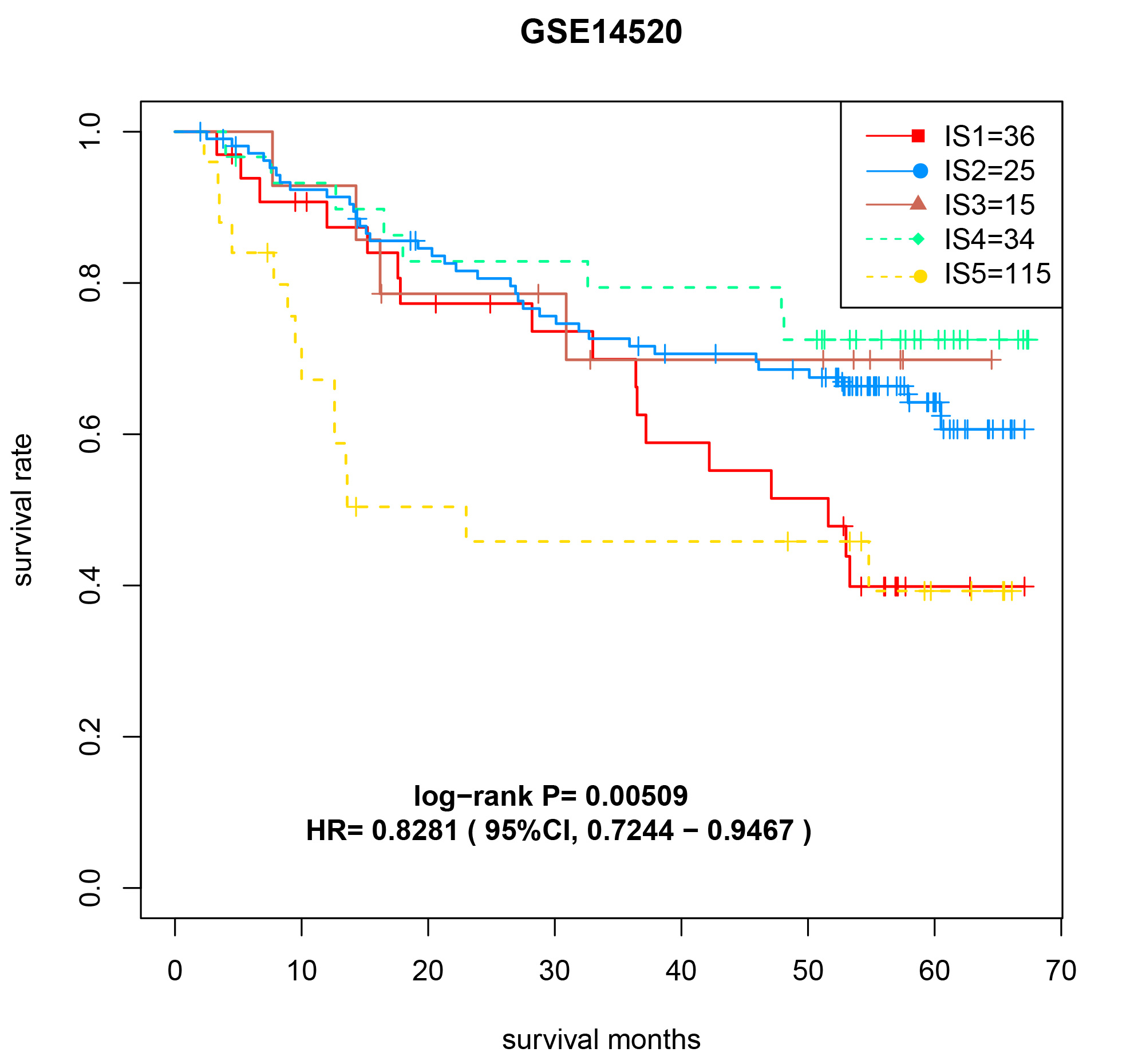

Supplement: Supplementary Materials — Figure S1. The prognosis of 5 immune subgroups was verified in validation cohort by KM analysis. Figure S2. The prognosis of 5 immune subgroups was verified in HBV-related and non-HBV-related HCC groups by KM analysis. Figure S3. The immune subgroups and gene modules in the HCC of the experimental cohort. Patients were arranged based on the predicted immune subgroups. Genes were ordered according to gene modules. Figure S4. The molecular characteristics of the immune subgroups. Figure S5. The distribution of 15 inferred immune compositions across 5 immune subgroups. Figure S6. The distribution of 7 inferred immune compositions across 5 immune subgroups. Figure S7. The depiction of the immune landscape of HCC from IS3-5. (A) The trajectory of development of 3 subtypes from IS3 based on the immune landscape, and (B) the distribution of 7 gene module patterns among 3 subtypes from IS3. Figure S8. The depiction of immune landscape of HCC from IS4. (A) The trajectory of development of 3 subtypes of IS4 based on the immune landscape, and (B) the distribution of 7 gene module patterns among 3 subtypes of IS4. Figure S9. Figure S9. The depiction of the immune landscape of HCC from IS5. (A) the trajectory of development of 3 subtypes from IS5 based on the immune landscape, and (B) the distribution of 7 gene module patterns among 3 subtypes from IS5. Figure S10. The verification by KM analysis of 3 subtypes from IS1 on validation cohort. Table S1. Univariate and multivariable Cox regression analyses of overall survival across clinical index and immune subtypes. Table S2. Validation of gene modules via univariate Cox analysis. [file 7253876.f1.zip › 7253876.f1/Figure S1 (1).jpg]

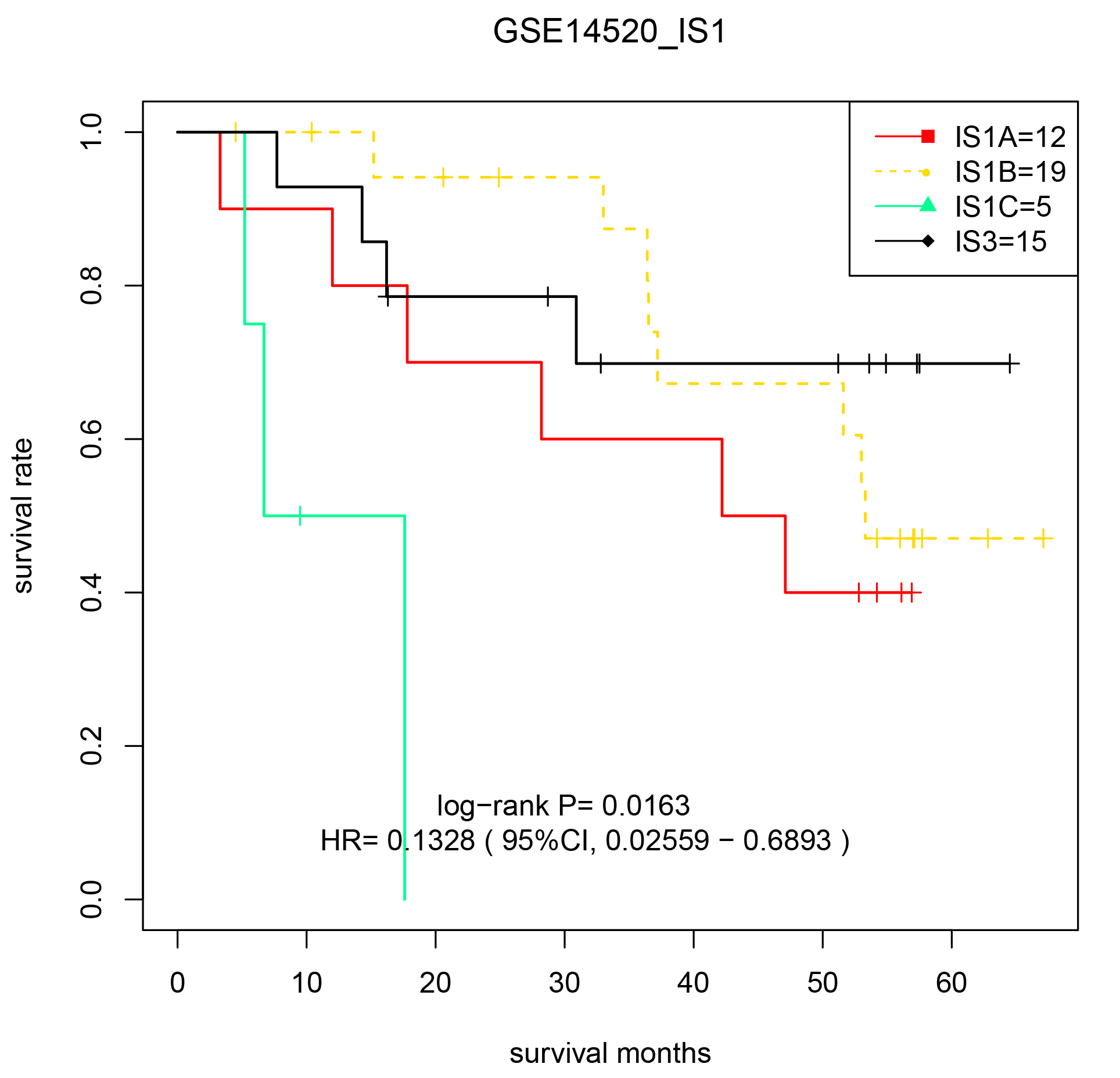

Supplement: Supplementary Materials — Figure S1. The prognosis of 5 immune subgroups was verified in validation cohort by KM analysis. Figure S2. The prognosis of 5 immune subgroups was verified in HBV-related and non-HBV-related HCC groups by KM analysis. Figure S3. The immune subgroups and gene modules in the HCC of the experimental cohort. Patients were arranged based on the predicted immune subgroups. Genes were ordered according to gene modules. Figure S4. The molecular characteristics of the immune subgroups. Figure S5. The distribution of 15 inferred immune compositions across 5 immune subgroups. Figure S6. The distribution of 7 inferred immune compositions across 5 immune subgroups. Figure S7. The depiction of the immune landscape of HCC from IS3-5. (A) The trajectory of development of 3 subtypes from IS3 based on the immune landscape, and (B) the distribution of 7 gene module patterns among 3 subtypes from IS3. Figure S8. The depiction of immune landscape of HCC from IS4. (A) The trajectory of development of 3 subtypes of IS4 based on the immune landscape, and (B) the distribution of 7 gene module patterns among 3 subtypes of IS4. Figure S9. Figure S9. The depiction of the immune landscape of HCC from IS5. (A) the trajectory of development of 3 subtypes from IS5 based on the immune landscape, and (B) the distribution of 7 gene module patterns among 3 subtypes from IS5. Figure S10. The verification by KM analysis of 3 subtypes from IS1 on validation cohort. Table S1. Univariate and multivariable Cox regression analyses of overall survival across clinical index and immune subtypes. Table S2. Validation of gene modules via univariate Cox analysis. [file 7253876.f1.zip › 7253876.f1/Figure S10 (1).jpg]

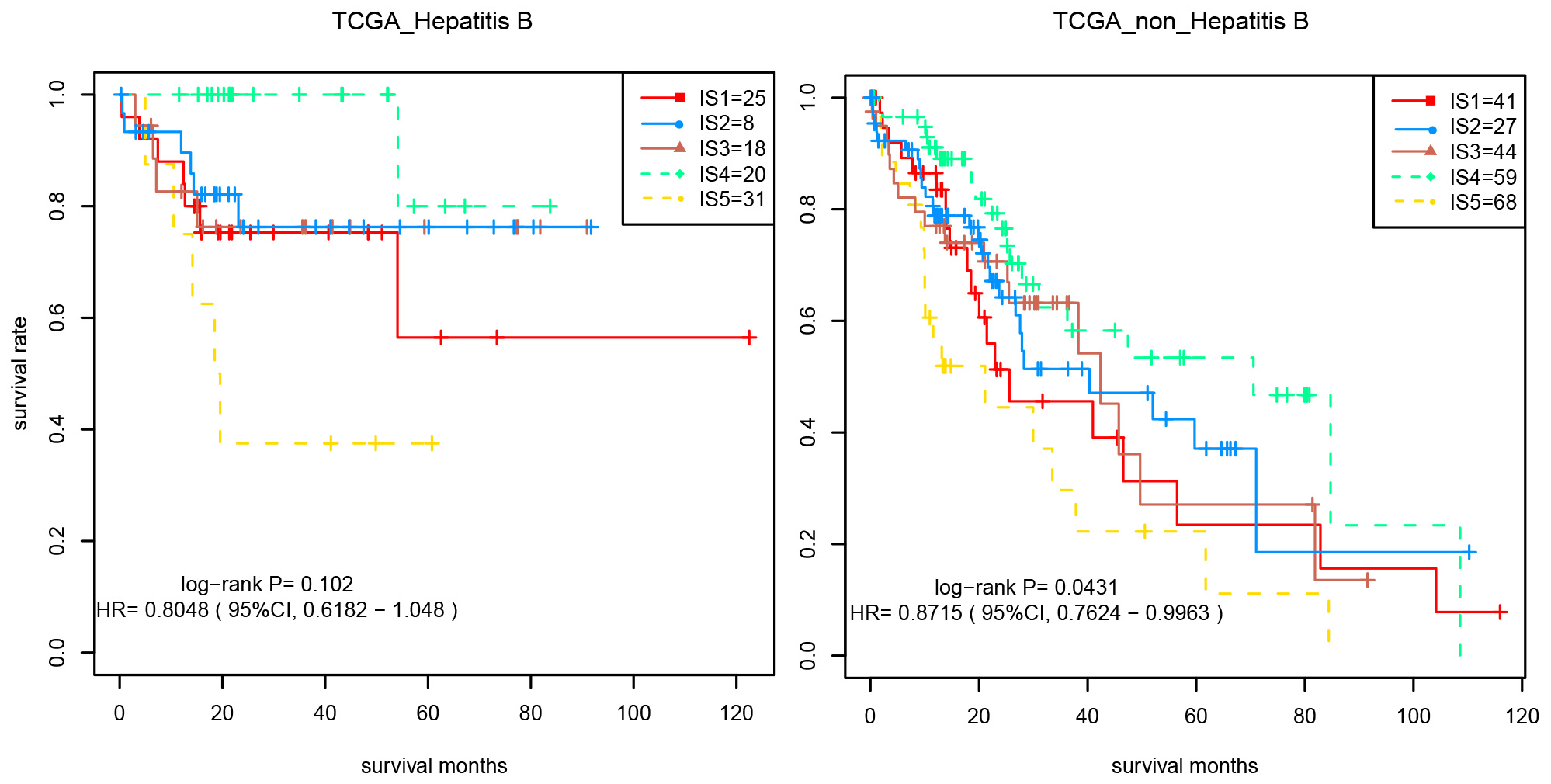

Supplement: Supplementary Materials — Figure S1. The prognosis of 5 immune subgroups was verified in validation cohort by KM analysis. Figure S2. The prognosis of 5 immune subgroups was verified in HBV-related and non-HBV-related HCC groups by KM analysis. Figure S3. The immune subgroups and gene modules in the HCC of the experimental cohort. Patients were arranged based on the predicted immune subgroups. Genes were ordered according to gene modules. Figure S4. The molecular characteristics of the immune subgroups. Figure S5. The distribution of 15 inferred immune compositions across 5 immune subgroups. Figure S6. The distribution of 7 inferred immune compositions across 5 immune subgroups. Figure S7. The depiction of the immune landscape of HCC from IS3-5. (A) The trajectory of development of 3 subtypes from IS3 based on the immune landscape, and (B) the distribution of 7 gene module patterns among 3 subtypes from IS3. Figure S8. The depiction of immune landscape of HCC from IS4. (A) The trajectory of development of 3 subtypes of IS4 based on the immune landscape, and (B) the distribution of 7 gene module patterns among 3 subtypes of IS4. Figure S9. Figure S9. The depiction of the immune landscape of HCC from IS5. (A) the trajectory of development of 3 subtypes from IS5 based on the immune landscape, and (B) the distribution of 7 gene module patterns among 3 subtypes from IS5. Figure S10. The verification by KM analysis of 3 subtypes from IS1 on validation cohort. Table S1. Univariate and multivariable Cox regression analyses of overall survival across clinical index and immune subtypes. Table S2. Validation of gene modules via univariate Cox analysis. [file 7253876.f1.zip › 7253876.f1/Figure S2 (1).jpg]

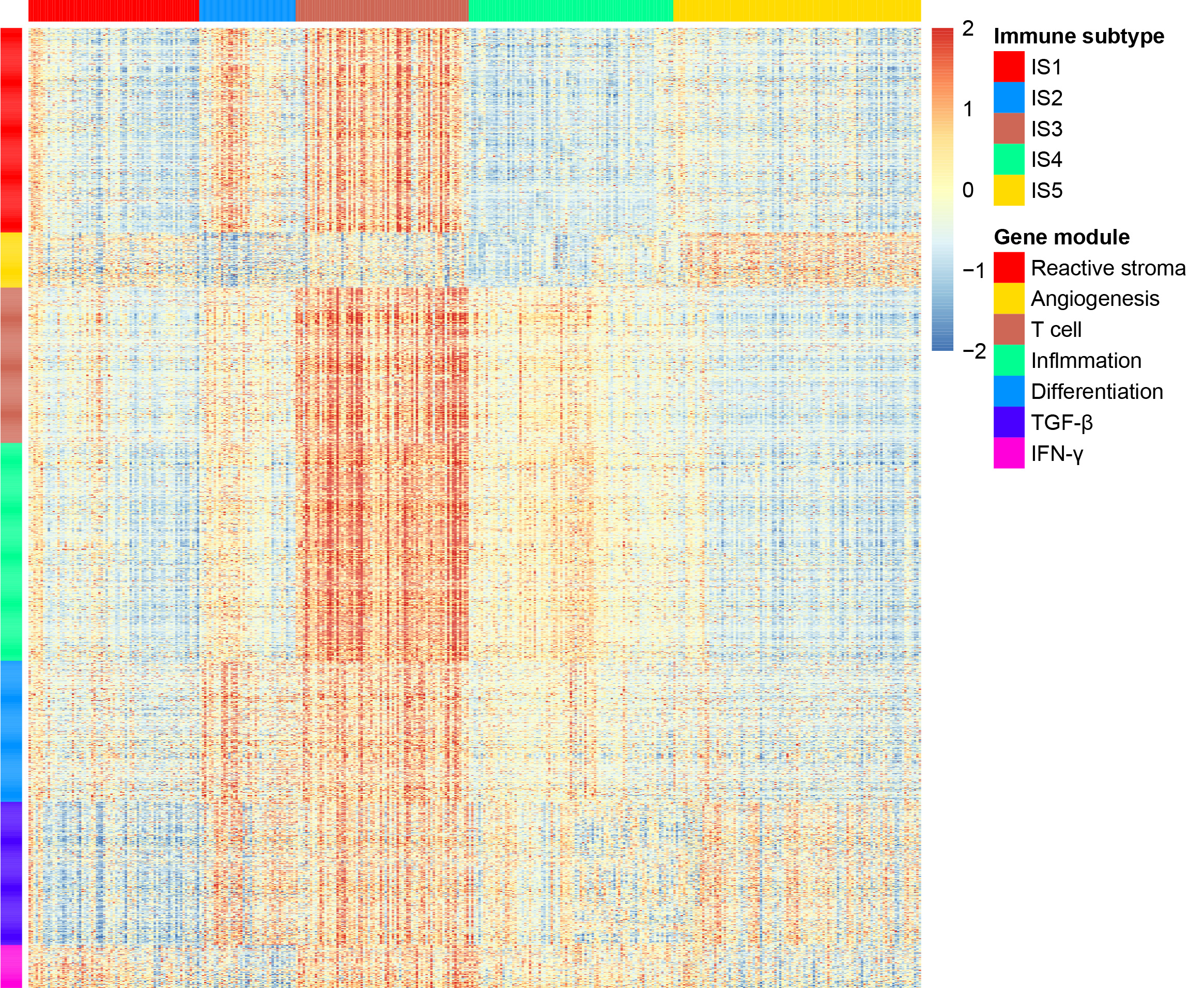

Supplement: Supplementary Materials — Figure S1. The prognosis of 5 immune subgroups was verified in validation cohort by KM analysis. Figure S2. The prognosis of 5 immune subgroups was verified in HBV-related and non-HBV-related HCC groups by KM analysis. Figure S3. The immune subgroups and gene modules in the HCC of the experimental cohort. Patients were arranged based on the predicted immune subgroups. Genes were ordered according to gene modules. Figure S4. The molecular characteristics of the immune subgroups. Figure S5. The distribution of 15 inferred immune compositions across 5 immune subgroups. Figure S6. The distribution of 7 inferred immune compositions across 5 immune subgroups. Figure S7. The depiction of the immune landscape of HCC from IS3-5. (A) The trajectory of development of 3 subtypes from IS3 based on the immune landscape, and (B) the distribution of 7 gene module patterns among 3 subtypes from IS3. Figure S8. The depiction of immune landscape of HCC from IS4. (A) The trajectory of development of 3 subtypes of IS4 based on the immune landscape, and (B) the distribution of 7 gene module patterns among 3 subtypes of IS4. Figure S9. Figure S9. The depiction of the immune landscape of HCC from IS5. (A) the trajectory of development of 3 subtypes from IS5 based on the immune landscape, and (B) the distribution of 7 gene module patterns among 3 subtypes from IS5. Figure S10. The verification by KM analysis of 3 subtypes from IS1 on validation cohort. Table S1. Univariate and multivariable Cox regression analyses of overall survival across clinical index and immune subtypes. Table S2. Validation of gene modules via univariate Cox analysis. [file 7253876.f1.zip › 7253876.f1/Figure S3 (1).jpg]

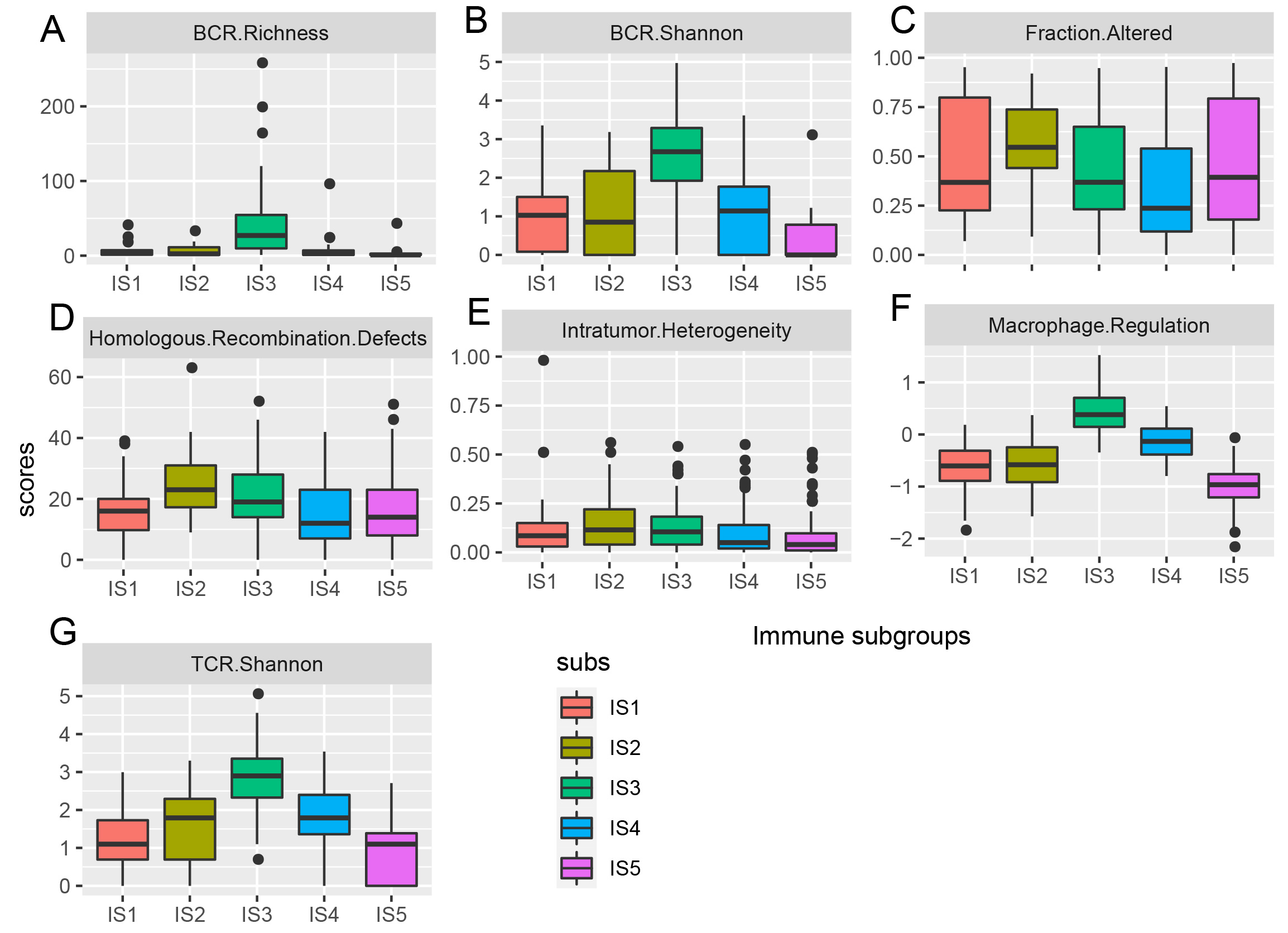

Supplement: Supplementary Materials — Figure S1. The prognosis of 5 immune subgroups was verified in validation cohort by KM analysis. Figure S2. The prognosis of 5 immune subgroups was verified in HBV-related and non-HBV-related HCC groups by KM analysis. Figure S3. The immune subgroups and gene modules in the HCC of the experimental cohort. Patients were arranged based on the predicted immune subgroups. Genes were ordered according to gene modules. Figure S4. The molecular characteristics of the immune subgroups. Figure S5. The distribution of 15 inferred immune compositions across 5 immune subgroups. Figure S6. The distribution of 7 inferred immune compositions across 5 immune subgroups. Figure S7. The depiction of the immune landscape of HCC from IS3-5. (A) The trajectory of development of 3 subtypes from IS3 based on the immune landscape, and (B) the distribution of 7 gene module patterns among 3 subtypes from IS3. Figure S8. The depiction of immune landscape of HCC from IS4. (A) The trajectory of development of 3 subtypes of IS4 based on the immune landscape, and (B) the distribution of 7 gene module patterns among 3 subtypes of IS4. Figure S9. Figure S9. The depiction of the immune landscape of HCC from IS5. (A) the trajectory of development of 3 subtypes from IS5 based on the immune landscape, and (B) the distribution of 7 gene module patterns among 3 subtypes from IS5. Figure S10. The verification by KM analysis of 3 subtypes from IS1 on validation cohort. Table S1. Univariate and multivariable Cox regression analyses of overall survival across clinical index and immune subtypes. Table S2. Validation of gene modules via univariate Cox analysis. [file 7253876.f1.zip › 7253876.f1/Figure S4 (1).jpg]

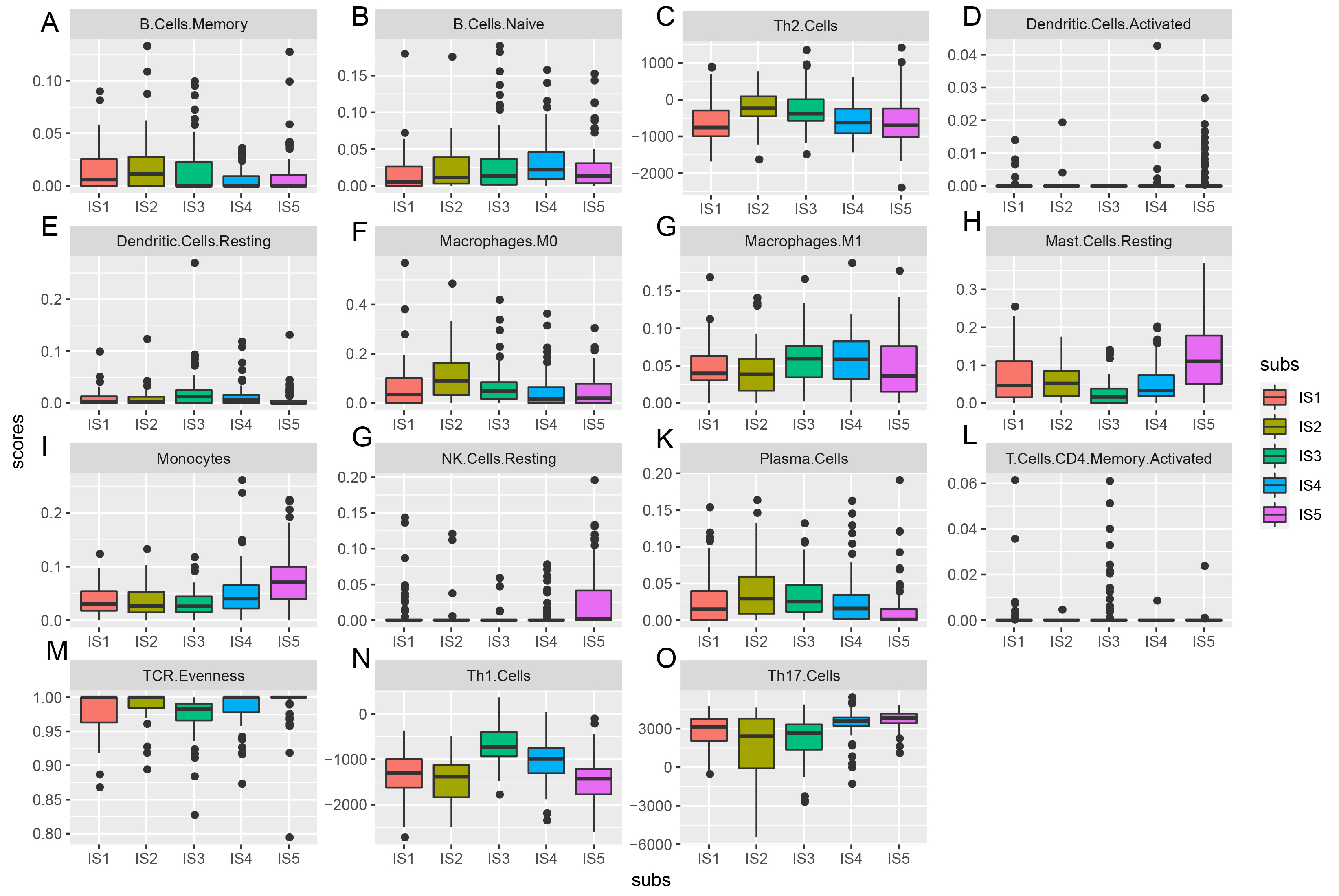

Supplement: Supplementary Materials — Figure S1. The prognosis of 5 immune subgroups was verified in validation cohort by KM analysis. Figure S2. The prognosis of 5 immune subgroups was verified in HBV-related and non-HBV-related HCC groups by KM analysis. Figure S3. The immune subgroups and gene modules in the HCC of the experimental cohort. Patients were arranged based on the predicted immune subgroups. Genes were ordered according to gene modules. Figure S4. The molecular characteristics of the immune subgroups. Figure S5. The distribution of 15 inferred immune compositions across 5 immune subgroups. Figure S6. The distribution of 7 inferred immune compositions across 5 immune subgroups. Figure S7. The depiction of the immune landscape of HCC from IS3-5. (A) The trajectory of development of 3 subtypes from IS3 based on the immune landscape, and (B) the distribution of 7 gene module patterns among 3 subtypes from IS3. Figure S8. The depiction of immune landscape of HCC from IS4. (A) The trajectory of development of 3 subtypes of IS4 based on the immune landscape, and (B) the distribution of 7 gene module patterns among 3 subtypes of IS4. Figure S9. Figure S9. The depiction of the immune landscape of HCC from IS5. (A) the trajectory of development of 3 subtypes from IS5 based on the immune landscape, and (B) the distribution of 7 gene module patterns among 3 subtypes from IS5. Figure S10. The verification by KM analysis of 3 subtypes from IS1 on validation cohort. Table S1. Univariate and multivariable Cox regression analyses of overall survival across clinical index and immune subtypes. Table S2. Validation of gene modules via univariate Cox analysis. [file 7253876.f1.zip › 7253876.f1/Figure S5 (1).jpg]

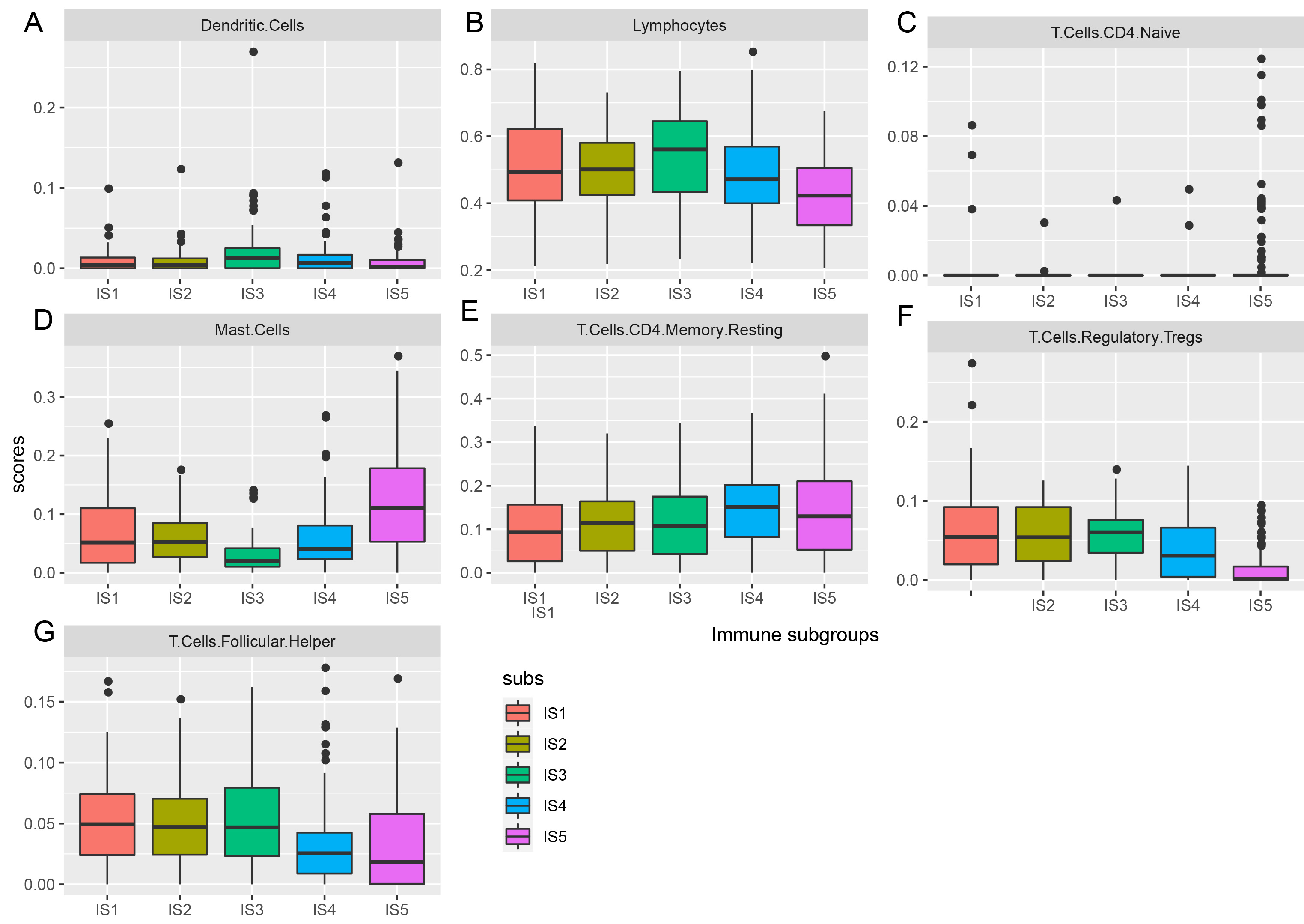

Supplement: Supplementary Materials — Figure S1. The prognosis of 5 immune subgroups was verified in validation cohort by KM analysis. Figure S2. The prognosis of 5 immune subgroups was verified in HBV-related and non-HBV-related HCC groups by KM analysis. Figure S3. The immune subgroups and gene modules in the HCC of the experimental cohort. Patients were arranged based on the predicted immune subgroups. Genes were ordered according to gene modules. Figure S4. The molecular characteristics of the immune subgroups. Figure S5. The distribution of 15 inferred immune compositions across 5 immune subgroups. Figure S6. The distribution of 7 inferred immune compositions across 5 immune subgroups. Figure S7. The depiction of the immune landscape of HCC from IS3-5. (A) The trajectory of development of 3 subtypes from IS3 based on the immune landscape, and (B) the distribution of 7 gene module patterns among 3 subtypes from IS3. Figure S8. The depiction of immune landscape of HCC from IS4. (A) The trajectory of development of 3 subtypes of IS4 based on the immune landscape, and (B) the distribution of 7 gene module patterns among 3 subtypes of IS4. Figure S9. Figure S9. The depiction of the immune landscape of HCC from IS5. (A) the trajectory of development of 3 subtypes from IS5 based on the immune landscape, and (B) the distribution of 7 gene module patterns among 3 subtypes from IS5. Figure S10. The verification by KM analysis of 3 subtypes from IS1 on validation cohort. Table S1. Univariate and multivariable Cox regression analyses of overall survival across clinical index and immune subtypes. Table S2. Validation of gene modules via univariate Cox analysis. [file 7253876.f1.zip › 7253876.f1/Figure S6 (1).jpg]

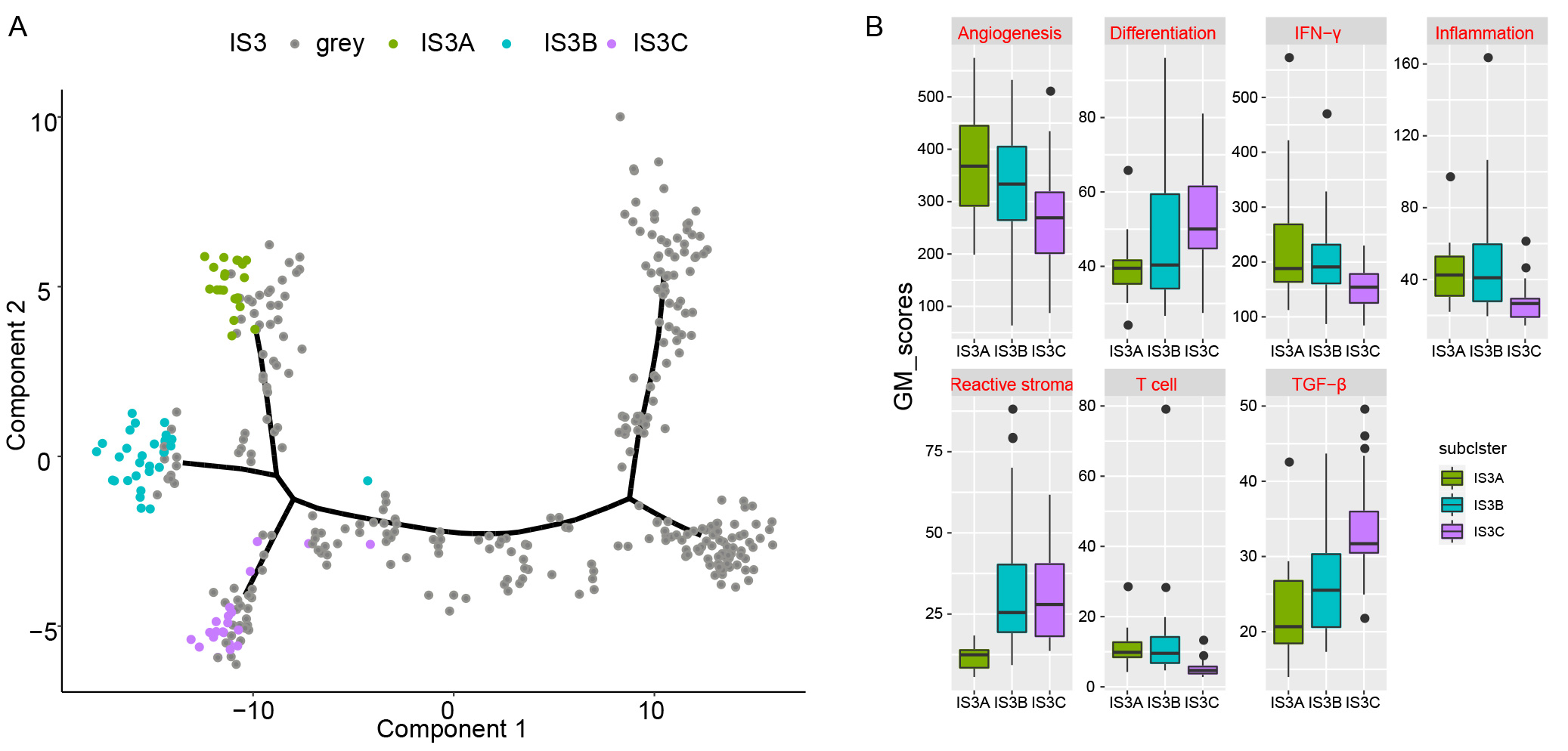

Supplement: Supplementary Materials — Figure S1. The prognosis of 5 immune subgroups was verified in validation cohort by KM analysis. Figure S2. The prognosis of 5 immune subgroups was verified in HBV-related and non-HBV-related HCC groups by KM analysis. Figure S3. The immune subgroups and gene modules in the HCC of the experimental cohort. Patients were arranged based on the predicted immune subgroups. Genes were ordered according to gene modules. Figure S4. The molecular characteristics of the immune subgroups. Figure S5. The distribution of 15 inferred immune compositions across 5 immune subgroups. Figure S6. The distribution of 7 inferred immune compositions across 5 immune subgroups. Figure S7. The depiction of the immune landscape of HCC from IS3-5. (A) The trajectory of development of 3 subtypes from IS3 based on the immune landscape, and (B) the distribution of 7 gene module patterns among 3 subtypes from IS3. Figure S8. The depiction of immune landscape of HCC from IS4. (A) The trajectory of development of 3 subtypes of IS4 based on the immune landscape, and (B) the distribution of 7 gene module patterns among 3 subtypes of IS4. Figure S9. Figure S9. The depiction of the immune landscape of HCC from IS5. (A) the trajectory of development of 3 subtypes from IS5 based on the immune landscape, and (B) the distribution of 7 gene module patterns among 3 subtypes from IS5. Figure S10. The verification by KM analysis of 3 subtypes from IS1 on validation cohort. Table S1. Univariate and multivariable Cox regression analyses of overall survival across clinical index and immune subtypes. Table S2. Validation of gene modules via univariate Cox analysis. [file 7253876.f1.zip › 7253876.f1/Figure S7 (1).jpg]

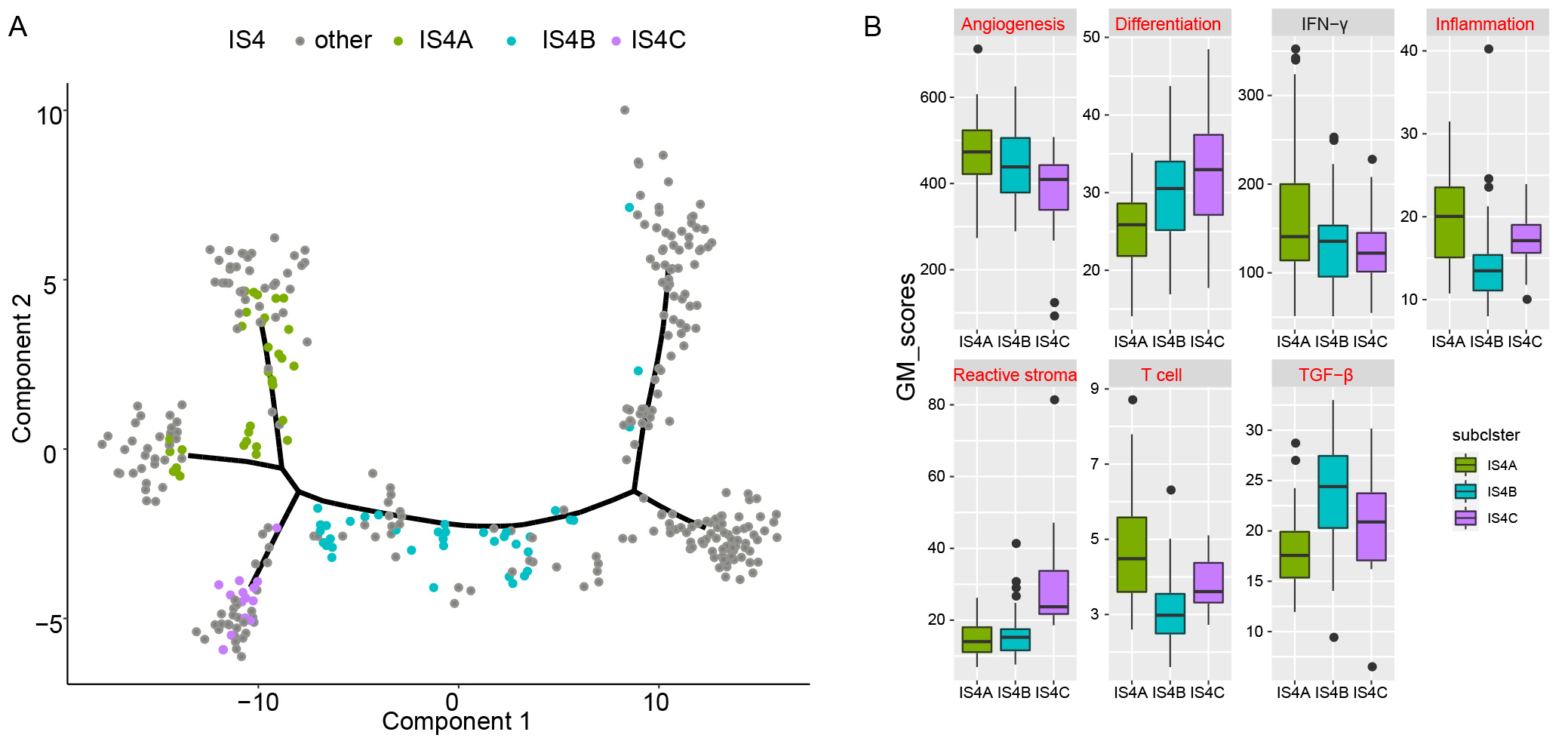

Supplement: Supplementary Materials — Figure S1. The prognosis of 5 immune subgroups was verified in validation cohort by KM analysis. Figure S2. The prognosis of 5 immune subgroups was verified in HBV-related and non-HBV-related HCC groups by KM analysis. Figure S3. The immune subgroups and gene modules in the HCC of the experimental cohort. Patients were arranged based on the predicted immune subgroups. Genes were ordered according to gene modules. Figure S4. The molecular characteristics of the immune subgroups. Figure S5. The distribution of 15 inferred immune compositions across 5 immune subgroups. Figure S6. The distribution of 7 inferred immune compositions across 5 immune subgroups. Figure S7. The depiction of the immune landscape of HCC from IS3-5. (A) The trajectory of development of 3 subtypes from IS3 based on the immune landscape, and (B) the distribution of 7 gene module patterns among 3 subtypes from IS3. Figure S8. The depiction of immune landscape of HCC from IS4. (A) The trajectory of development of 3 subtypes of IS4 based on the immune landscape, and (B) the distribution of 7 gene module patterns among 3 subtypes of IS4. Figure S9. Figure S9. The depiction of the immune landscape of HCC from IS5. (A) the trajectory of development of 3 subtypes from IS5 based on the immune landscape, and (B) the distribution of 7 gene module patterns among 3 subtypes from IS5. Figure S10. The verification by KM analysis of 3 subtypes from IS1 on validation cohort. Table S1. Univariate and multivariable Cox regression analyses of overall survival across clinical index and immune subtypes. Table S2. Validation of gene modules via univariate Cox analysis. [file 7253876.f1.zip › 7253876.f1/Figure S8 (1).jpg]

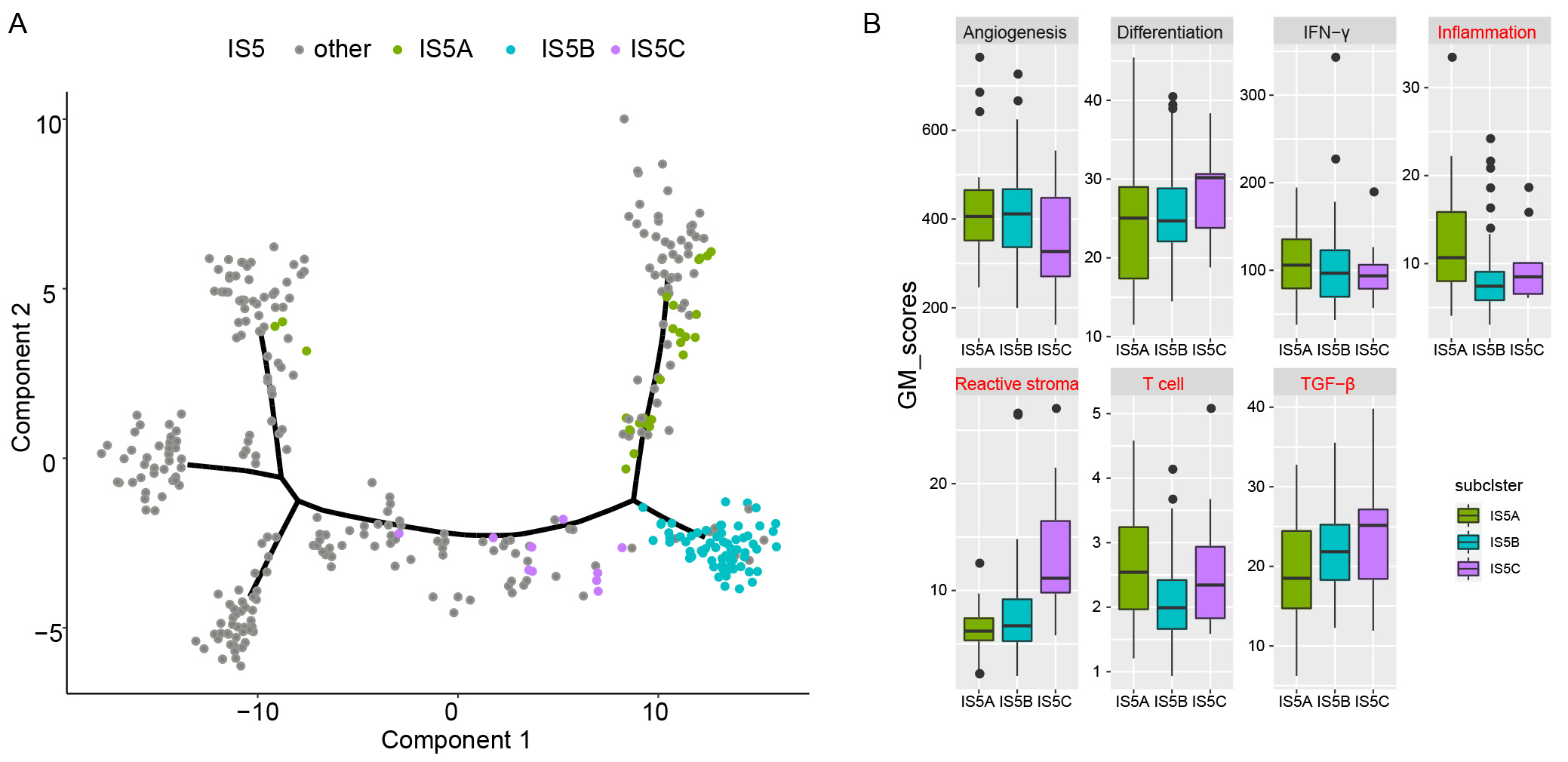

Supplement: Supplementary Materials — Figure S1. The prognosis of 5 immune subgroups was verified in validation cohort by KM analysis. Figure S2. The prognosis of 5 immune subgroups was verified in HBV-related and non-HBV-related HCC groups by KM analysis. Figure S3. The immune subgroups and gene modules in the HCC of the experimental cohort. Patients were arranged based on the predicted immune subgroups. Genes were ordered according to gene modules. Figure S4. The molecular characteristics of the immune subgroups. Figure S5. The distribution of 15 inferred immune compositions across 5 immune subgroups. Figure S6. The distribution of 7 inferred immune compositions across 5 immune subgroups. Figure S7. The depiction of the immune landscape of HCC from IS3-5. (A) The trajectory of development of 3 subtypes from IS3 based on the immune landscape, and (B) the distribution of 7 gene module patterns among 3 subtypes from IS3. Figure S8. The depiction of immune landscape of HCC from IS4. (A) The trajectory of development of 3 subtypes of IS4 based on the immune landscape, and (B) the distribution of 7 gene module patterns among 3 subtypes of IS4. Figure S9. Figure S9. The depiction of the immune landscape of HCC from IS5. (A) the trajectory of development of 3 subtypes from IS5 based on the immune landscape, and (B) the distribution of 7 gene module patterns among 3 subtypes from IS5. Figure S10. The verification by KM analysis of 3 subtypes from IS1 on validation cohort. Table S1. Univariate and multivariable Cox regression analyses of overall survival across clinical index and immune subtypes. Table S2. Validation of gene modules via univariate Cox analysis. [file 7253876.f1.zip › 7253876.f1/Figure S9 (1).jpg]
